# Immune and genomic signatures in oral (head and neck) cancer

**DOI:** 10.1016/j.heliyon.2018.e00880

**Published:** 2018-11-02

**Authors:** Prasenjit Chakraborty, Tanusri Karmakar, Neeraj Arora, Geetashree Mukherjee

**Affiliations:** Department of Histopathology, Tata Medical Center, Kolkata, India

**Keywords:** Oncology, Cancer research, Immunology, Genetics

## Abstract

Head and neck squamous cell carcinoma (HNSCC) is responsible for a large number of deaths each year. Oral cancer is the most frequent subtype of HNSCC. Historically, oral cancer has been associated with an increase in the consumption of tobacco and alcohol products, seen especially in the Asian subcontinent. It has also been associated with infection by the human papilloma virus (HPV), particularly strain HPV16. Treatment usually involves a multidisciplinary approach of surgery combined with chemotherapy and radiation. The advent of immunotherapy has broadened the scope for treatment. A better immune response to the tumour can also elicit the action of other therapeutic approaches. A heightened immune response, on the other hand, can lead to resistant tumour formation through the process of immunoediting. Molecular profiling of the tumour microenvironment (TME) can provide us with better insight into the mechanism and progression of the disease, ultimately opening up new therapeutic options. High-throughput molecular profiling techniques over the past decade have enabled us to appreciate the heterogeneity of the TME. In this review, we will be describing the clinicopathological role of the immune and genomic landscape in oral cancer. This study will update readers on the several immunological and genetic factors that can play an important function as predictive and prognostic biomarkers in various forms of head and neck cancer, with a special emphasis on oral carcinoma.

## 1 Introduction

Immunotherapy in the field of oncobiology has been rapidly advancing over the past decade. The role of tumour-infiltrating lymphocytes (TILs) in the tumour microenvironment (TME) gives a predefined definition of cancer being an immunological disease apart from its genetic basis [1, 2, 3]. Tumours have the ability to organize an immunosuppressive microenvironment that is dependent on the reciprocal interactions between the tumour and its host. Generally, the released tumour antigens are processed and presented by antigen-presenting cells (APCs) to the effector T cells. The effector T cells, thus activated, can mount an antitumour response [4, 5]. Tumour cells develop resistance via induced T cell tolerance, leading to an immune escape condition due to alterations in immunity. This creates an immunosuppressive environment through complex signalling between tumours and their associated cells.

Head and neck squamous cell carcinoma (HNSCC) represents cancer of the squamous cells that line the moist, mucosal surfaces inside the head and neck. It includes the oral cavity, pharynx, larynx, salivary glands, paranasal sinuses and nasal cavity. HNSCC is the most aggressive malignant neoplasm arising in the mucosa of the upper aerodigestive tract [6]. Patients suffering from HNSCC have myriad alterations in their immune cell population. They demand a potential therapeutic approach to be highlighted for the proper recovery and lower recurring outcomes. HNSCC immunobiology is essentially linked to the immune system of the host. Immunological surveillance is a monitoring process of the immune system to detect and destroy invading pathogens and neoplastically transformed cells in the body. The escape of the tumour-associated antigens (TAA) from host immunity signifies a failure of immune surveillance to control tumour progression. Although molecular and cellular immunology has gained immense advances in the last few decades, the biotherapy of cancer, including antitumour vaccines, still needs a satisfactory outcome. The major reason for this drawback is that tumours in general, especially HNSCC, can evade and escape host immunity through multiple strategies. Tumours are also known to express immunoinhibitory molecules [7, 8, 9, 10] and, as a result, both the local and systemic immunity are suppressed. Finally, in advanced HNSCC death of the immune effector cells is profound [11]. The mechanisms for immune suppression are yet to be revealed and several large investigations are already in progress.

Alterations of the genome are among the main factors that have been implicated in the etiology of cancer. These alterations can be observed as the insertion, deletion or substitution of nucleotides or chromosomal abnormalities leading to the manifestation of a defective phenotype [12, 13, 14]. Therefore, genomic biomarkers can be very useful to predict changes in tumour biology in response to treatment and can be potential therapeutic targets. The presence of oncogenes and their expression at very high levels contribute to tumourigenesis. In the case of tumour suppressor genes, the scenario is exactly the opposite, and a loss of expression is generally associated with progression to cancer. The genes involved and their degree of change in expression varies according to different tumour types. Several techniques have been employed to pinpoint the genetic instabilities taking place inside a tumour cell, which can help to characterize different subsets of tumours. Approaches ranging from oncogene arrays to comparative genome hybridization (CGH), BAC end sequencing and quantitative microsatellite analysis have provided oncology researchers with useful experimental strategies for mapping the genomic markers involved in oncogenesis [15, 16]. Modern techniques such as RNA-seq technology have been successfully used to identify global gene expression patterns in tongue squamous cell carcinoma [17]. This RNA-seq study identified several genetic alterations that can explain the highly invasive behavioural pattern of tongue squamous cell carcinoma.

This review aims to identify the mechanisms involved in the immunosuppression of HNSCC biology and to demonstrate a few experimental and therapeutic approaches implemented to surmount the tumour associated immune dysfunction in HNSCC. Critical genetic signatures have also been described in detail, which are known to play an important role in the development of oral cancer. This would finally be supportive for the proper developmental immunotherapy approaches.

## 2 Main text

### 2.1 Immune markers/profile in HNSCC

In HNSCC, an increase in regulatory T cells (Tregs) and myeloid-derived suppressor cells (MDSCs), as well as a decrease in the absolute number of T cells and CD8^+^ effector T cell dysfunction, has already been reported [18, 19, 20]. This profiling of the immune cells can correlate to the treatment efficacy and prediction of prognosis. Activation of T cells requires two simultaneous signals from APCs. The ‘first signal’ is the direct interaction of the T cell receptor (TCR) on a T cell with the major histocompatibility complex (MHC) on APCs. The second is the ‘co-stimulatory signal’ that aids in the interaction of B7 on the APC surface to CD28 on the T cell [3]. A third signal made up of immune-activating cytokines such as IL-12 and type I (IFNα/β) or type II (IFNγ) interferon [21] must be involved in the context of the previous two signals.

Recently, researchers have been investigating the expression of tumour infiltrating lymphocytes (TILs), including T cells, B cells, macrophages and neutrophils in solid tumours [22, 23]. Tregs in general control the autoreactive lymphocytes, but they can also downregulate the immune system response to tumour-associated antigen (TAA). In many solid tumours these Tregs are found especially in the TILs as well as in peripheral circulation [24, 25, 26, 27]. Although, the reason for their accumulation is still not clear. The enrichment of Tregs in tumours could be induced by the conversion of FOXP3^-^ T cells into FOXP3^+^ T cells in the presence of TGFβ1 and retinoic acid [28, 29]. Inversely, in tumour sites, the CD44^hi^ memory Tregs specific for self-antigen are expanded and that protects the tumour against the immune response of the host [30]. Hence, the conclusion can be drawn that Treg activation and expansion takes place in the TME. In patients with HNSCC, severe immune disruption is found; hence, the antitumour response is impaired [31], but few effector immune cells are reported to have favourable outcome [32].

A strong correlation between the density and location of TILs may lead to better prognosis of cancer and can be implemented in quantitative pathology. The ‘immunoscore’ is one such method based upon a standardized algorithm, where CD3^+^ and CD8^+^ TILs are quantified in the tumour core (CT) and invasive margin (IM). This proved to be an established prognostic tool in colorectal cancer [33, 34].

### 2.2 Role of immune marker expression in tumour progression

Subsets of immune and non-immune cells interact with each other and are required for an efficient functioning of the immune system. Leukocyte migration and trafficking to their destined tissues or sites is specially regulated by chemokines. Chemokines are mostly produced during infection or inflammation, except for CCL19 and CCL21, which are constitutively produced and control cell movement during homeostasis [35]. All these interactions are important for protective immunity but peripheral tolerance and regulations by Tregs are also essential. On the basis of these discoveries, we will discuss the involvement of the most significant signature markers and their role in immune development.

#### 2.2.1 CCR7

CC-chemokine receptor 7 (CCR7) is expressed by different immune cell subsets [36] and is mainly involved in the homing (through its ligands CCL19 andCCL21) of many T cell subpopulations and antigen presenting dendritic cells (DC) to the lymph node. CCR7 is an indispensable mediator for tranquil thymic T cell development and behaves as negative selection for self-reactive T cells. A seven-transmembrane-spanning domain protein that mediates its signal through heterotrimeric G proteins and is generally involved in the homing of the immune cells to their secondary lymphoid organs and positioning them to defined functional compartments. They are expressed by a number of cells such as semi-mature and mature DCs [37], thymocytes during defined stages of their development [38], naïve B and T cells [39, 40], Tregs [41] and a subpopulation of memory T cells known as central memory T (TCM) cells [40]. CCR7 is critical in its functional status at subcellular level and its expression on transformed and metastatic cells has been reported in different cancer types. CCR7 in combination with CD45RA^+^ (naive) and CD45RA^−^ (memory) subtypes can be sorted into: CD45RA^−^CCR7^+^ (central memory), CD45RA^−^CCR7^−^ (effector/memory), CD45RA^+^CCR7^−^ (terminally differentiated). CD45RA^−^CCR7^−^ effector memory subtypes are mostly increased in the invading TILs that could significantly influence their fate in comparison to conventional T cells, indicating immunosuppressive consequences in gastric cancers [42]. It has been reported that invasive breast cancer cells may undergo targeted migration towards lymphatics induced by tumour cell expression of CCR7 bound to its ligands called the lymphoid homing chemokines CCL19/CCL21. Hence, CCR7 play crucial role in lymphatic migration of tumour cells similarly to lymph nodes draining dendritic cell immigration. This can be also explained as a hijack of physiological chemokine driven leucocyte migration by CCR7 in lymphatic migration of tumor cells during metastasis. This highlights the significant role of tumour microenvironment during immune cell mediated inflammation in determining the repertoire of expression of chemokines on immune cell infiltrate and developing tumour [43]. Although these facts indicate an abundant chemokine expression within the tumour mass to retain immune cells, but expression on tumour-associated leucocytes in its microenvironment still needs a global informative panel designing research on immune subset analysis. In HNSCC, elevated CCR7 expression is found to correlate with lymph node metastasis and tumour tissue histological differentiation status [44]. Tongue squamous cell carcinoma tissues and different cell lines detected the expression of CCR7 when compared to normal oral mucosa with no CCR7 expression [45]. With many such similar reports, CCR7 may be conferred to play an important role in predicting metastasis and prognosis in HNSCC patients.

#### 2.2.2 T cell

Naive T (T_N_) cells harbouring a given epitope specificity are released from the thymus as mature cells after following either a positive or a negative selection. T_N_ cells proliferate and differentiate into effector cells after a cognate antigen (Ag) encounter. Most of them migrate to peripheral tissues and infected sites to facilitate their destruction [46]. After Ag clearance, effector cells die, and few develop into long-lived memory T cells.

The development of high-throughput technologies such as multiplexing and flow cytometry has paved a unique contribution to immunology. With the use of monoclonal antibodies and spectral immunohistochemistry, phenotypic identification and characterization of a heterogeneous population of cells could be quantified using conjugated fluorochromes. Although, identification of different subsets of lymphocytes and other cells under flow cytometry can be done with a designed panel of eight colour fluorochrome conjugated antibodies (Table 1), the simultaneous detection of upto seventeen different markers is possible [47]. T_N,_ T central memory (T_CM_) and T effector memory (T_EM_) cells could be separated on the basis of expression of CD45, CD45 RA and CD45 RO, respectively [48]. At the single cell level both the phenotype and functional properties of T cells could also be recognized. For instance, CD27 (a member of the tumour necrosis factor receptor-TNF superfamily) and CCR7 both mediate homing and can be used in combination with CD45 RA to define the phenotypical subsets of CD8^+^ memory cells on the effector function basis. Antigen-specific T cell activation is mainly mediated by the human leukocyte antigen HLA-DR. Low HLA-DR expression significantly indicates impaired host defence [49, 50]. Among the cytolytic molecules, Granzyme (Gr) A and B and perforin are the most common. Gr B production depends on the production of Gr A. Henceforth, perforin-positive cells that are positive for both Gr A and B can be a good choice of marker for the assessment of cytological property.

**Table 1:** Flow cytometry panel design with 8 colour fluorochromes for immune subset analysis includes lymphocyte (T & B), monocytes, macrophages and dendritic cells in resected tumour samples from HNSCC patients and matched peripheral blood samples.

Cell Type	Tube	BV421	V500/BV510	FITC/BB 515	PE/PE-CF594	PerCp-Cy5.5	PE-Cy7	APC/Alexa647	APC-H7
T cell	T1	CD45RA	CD3	CD8	CCR7	CD4	CD45RO	CD38	HLA-DR
	T2	Perforin	CD3	CD8	Granzyme B	CD4	CD16 + CD56	CD103	-
	T3	GARP	CD3	CD8	LAP	CD4		CD278	-
	T4	TIM-3	CD3		CTLA4	CD4	PD-1	PDL-1	-
Treg	T1		CD3	CD25	FoxP3	CD4		CD127	-
B cell	T1	CD24	CD138	CD27	CD10	CD19	IgD	CD38	CD20
MDSC, Monocyte, DC	T1	CD200	CD3	CD16	CD1c	CD11c	CD14	CD169	HLA-DR
	T2	CD141	-	CD15	-	CD11c	-	CD123	HLA-DR
Macro-phage	T1	CD40	HLA-DR	CD206	CD81	-	CD33	CD192	CD11b
	T2	CD141	CD86	CD163	CD80	-	PD-1	PDL-1	CD11b

#### 2.2.3 Tregs

Suppressor Tregs that prevent autoimmunity contain a subpopulation of CD4^+^ T cells and have CD25, CTLA4, and CD39 marker expression [51, 52]. Tregs have multiple functions, including promoting cancer progression by causing anergy, apoptosis and cell cycle arrest of activated T cell with the production of IL-10, TGF-β and direct cell-cell contact. The inhibitory action of Tregs is also applied to DCs, natural killer (NK) and B cells [53, 54]. In HNSCC, an increased Treg population is found in the peripheral blood and T cell infiltrating tumour, suggesting an immune-suppressed status [55, 56, 57, 58]. Indoleamine 2, 3-dioxygenase (IDO) activity in antigen-presenting cells (APCs) can be induced by Tregs through CTLA-4 and CD80/CD86 interaction. Tryptophan is generally catabolized by IDO and its supply to immune cell is depleted, leading to an inhibition of effector T and NK cell functions [59, 60]. A recent study found a gene signature of 32 genes in Treg enriched tumours that are likely to be associated with favourable prognosis [61]. Two genes have been selected for special mention. The *TRAF3IP1* gene expresses the TRAF3IP1 protein, which interacts with another protein, TRAF3, to inhibit the type I interferon response. As expected, lower expression of this gene has been associated with a favourable outcome. Another gene, *LTB*, coding for the lymphotoxin beta has been shown to be elevated in patients showing a better response to therapy. The role of lymphotoxin beta is to stimulate Tregs so that they migrate away to lymph nodes from the tissue. Although these data point toward the immunosuppressive role of Tregs within the tumour microenvironment, it requires further validation through *in vivo* studies.

On the other hand, it has been found that after treatment Treg frequency gets elevated in HNSCC patients, indicating a correlation between oncologic treatment and Treg elevation. In several solid tumours, the role of FOXP3^+^ is associated with favourable outcomes. Data from 278 patients' formalin-fixed paraffin-embedded (FFPE) samples suggest that elevated FOXP3^+^ cells are associated with favourable prognosis and positively correlated to superior loco regional control [62, 63]. Thus, it seems that the opinion on the clinical relevance of intratumoural Tregs can be polarized. The heterogeneous property of Tregs can be influenced by the tumour site, molecular subtype and tumour stage. Indeed, biomarkers are not reliable indicators of the functional capacity of Tregs, since Tregs found in TME and that circulating to the periphery may not be the same in their functional repertoire [64]. The origin and phenotypic characteristics of Tregs that infiltrates human tumours are yet to be unfolded. Enhancement of Tregs can be beneficial to some patient groups while being detrimental to others. Additional studies are required to better understand the myriad roles of Tregs in the TME [65, 66].

#### 2.2.4 MDSCs

Myeloid-derived suppressor cells (MDSCs) are emerging as important markers of the myeloid cell lineage and play a major role in tumour-mediated immunosuppression [67]. In healthy individuals, immature myeloid cells in the bone marrow differentiate into mature granulocyte, macrophages or dendritic cells. However, in pathological conditions such as cancer, a block during differentiation leads to an accumulation of the population. At this stage, they lack the expression of markers for monocytes, macrophages and dendritic cells. Monocytes are one of the myeloid-derived cell types that have different expression of CD markers on their surface, especially CD14 and CD16, and can differentiate into both macrophages and dendritic cells. Both macrophages and dendritic cells play a crucial role in disease pathogenesis, including cancer [68, 69, 70, 71]. MDSCs can migrate to the tumour site, upregulating expression of arginase1 and iNOS (induced nitric oxide synthetase) but downregulating production of reactive oxygen intermediates (ROS), and/or can be rapidly differentiated to tumour associated macrophages (TAMS) [72, 73]. Cytokines are produced by the TAMS, which can induce T cell suppression non-specifically. Tumour-associated neutrophils (TANS), like TAMS, have distinct activation and differentiation states, and they develop a pro-tumourigenic phenotype largely driven by the presence of TGF-β [74]. The depletion of TANS reduces tumour growth and inhibits immunosuppression in the tumour microenvironment, thus leading to increased CD8^+^ cytotoxic T lymphocytes.

MDSCs are responsible for angiogenesis in HNSCC, and inhibition of the JAK/STAT pathway has been shown to reduce both MDSCs and angiogenesis [75]. Alterations of myelopoiesis-associated tumour growth leads to the recruitment of immunosuppressive MDSCs. Hence, MDSCs are induced by markers (TGFβ, VEGF and IL-6) associated with inflammation [76]. MDSCs isolated from some ovarian cancer patients have been found to exhibit hypermethylation [77]. Prostaglandin-E2 (PGE2)-induced upregulation of DNA methyltransferase 3A (DNMT3A) is responsible for the observed hypermethylation, which is also replicated in *in vitro* models. This MDSC-specific methylation is responsible for the downregulation of *S1PR4*, *RUNX1*, *AQP9*, *LMO2* or *FYN* genes. Most of these genes encode factors to prevent the suppressive activity of MDSCs. Hence, characterization of myeloid gene hypermethylation mediated by DNMT3A under the induction of PGE2 can be implemented in their identification under different inflammatory perspectives. It can also be a useful target for therapeutic intervention.

#### 2.2.5 Immune checkpoint molecules

Activated immune cells express some inhibitory checkpoint receptors (ICRs) on their surface. The receptors may be cytotoxic T lymphocyte-associated antigen 4(CTLA-4), programmed cell death-1(PD-1), T-cell immunoglobulin and mucin protein-3 (TIM-3) and lymphocyte activation gene-3 (LAG-3) that play an important role in the TME [3, 78]. Activated CD8^+^ T cells, NK cells, B cells, monocytes, and DCs express PD-1, a cell surface protein that, when bound by its ligand PD-L1, provides an inhibitory signal. The upregulation of PD-1 may even cause T cell exhaustion and tolerance [79]. Exhausted T cells can also be marked with another inhibitory receptor such as TIM-3, which is selectively expressed on activated interferon gamma (IFN-γ) produced by CD4^+^ and CD8^+^ T cells. Autologous TIL and peripheral blood samples may be compared for intratumoural and circulating immune cell expression, and then correlating them with the ICR expression would result in their expansion and/or suppression property in the TME.

### 2.3 Genomic signatures

Band q13 of chromosome 11 in humans harbours many candidate genes implicated in oral cancer. Several genetic drivers that function to confer a malignant phenotype to the host cell have been mapped to this locus [80]. The specialty of this locus is that a set of neighbouring gene are often co-amplified. It has been observed that amplification of this particular chromosomal band is often associated with worse prognosis during the investigation of cell lines obtained from oral cancer patients [81]. Within the 11q13.2-q13.4sub-region, an amplicon core has been identified that is responsible for oral squamous cell carcinoma (OSCC). This amplicon shows a high degree of complexity in both structure and composition [82]. Discontinuities are observed, suggestive of preferential re-amplification or counter selection. A study by Natrajan et al. [83] has shown that it is possible for an amplicon to be associated with more than one amplicon driver. By limiting candidates to the smallest regions of amplification, many drivers associated with this amplicon have been identified. It is quite possible that the observed phenotypes in oral cancer are a result of a co-operative interaction between the genetic markers of locus 11q13.

*CCND1* (encodes cyclin D1 that promotes the G1-S phase transition during the cell cycle) and *CTTN* (coding for cortactin, an F-actin binding protein that permit various protein interactions) are two of the most frequently associated drivers of locus 11q13. The amplification of *CTTN* confers resistance to the anticancer drug gefitinib, while amplification of *CCND1* is associated with resistance to cisplatin [84]. The presence of these oncogenes influencesthe clinicopathological characteristics, such as lymph node involvement, poor tumour differentiationand low survival, observed in OSCC [85]. Strong expression of cortactin is a predicting factor for increased cancer risk in oral premalignant lesions [86]. Epigallocatechin-3 gallate, an active polyphenol component of green tea, has various beneficial biological effects. Actin cytoskeleton remodelling through activation of FAK/Src signalling and a decrease in cortactin phosphorylation due to epigallocatechin-3 gallate treatment resulted in inhibition of oral cancer cell invasiveness and motility [87]. Expression levels of the *CCND1* gene could be used as a biomarker to select oral cancer patients who could benefit from induction chemotherapy. Although, lower cyclin D1 expression in the pretreatment biopsy samples is generally considered a favourable prognostic biomarker, *Zhong* et al. found that the higher the expression of cyclin D1 is, the better is the response of the patients towards chemotherapy [88]. In synergy with *CCND1*, overexpression of the *PPP1CA* gene, coding for the catalytic subunit of protein phosphatase 1α (PP1α), is responsible for the progression of oral cancer [89]. The RNA-binding protein quaking 5 (QKI-5) regulates the growth of the human oral cancer cell line CAL-27. This regulative function is attributed to its ability to regulate the mitogen-activated protein kinase (MAPK) pathway, leading to decreased expression of cyclin D1 [90]. The antagonistic effect of FOXO3a on the phosphoinositide-3-kinase (PI3K)/Akt pathway can also regulate the level of cyclin D1 in OSCC [91]. For this reason, FOXO3a activation has even been proposed as a therapeutic strategy for OSCC.

### 2.4 Markers located on other loci

Apart from 11q13, a gain of chromosome 8q has also been implicated in the development of OSCC [92]. A majority of structural rearrangements observed are isochromosomes or whole arm translocations. *FAK/PTK2* is one of the most important genes located within the chromosomal region 8q23–24, encoding the intracellular protein focal adhesion kinase (FAK) located in cellular structures called “focal adhesions”. Overexpression of this particular protein was detected in premalignant oral mucosa lesions at early stages of oral tumourigenesis [86]. The frequency of amplification of this particular locus increases with the grade of dysplasia. It is an important determinant in the pathogenesis, development and progression of a significant subset of oral squamous cell carcinomas. OSCC had higher frequencies of gain of chromosome arms 1q and 8q and chromosome region 11q1, and loss of genetic material from 3p and 8p2 [92].

### 2.5 Genes involved in oral carcinoma of the tongue and gingivo-buccal

In oral carcinoma located in the tongue, the expression of FADD (Fas-associated death domain-containing protein) was higher than that in adjacent areas [93]. This phenomenon can be attributed to genomic amplification in 11q13.3, impacting the regulation of the *FADD* gene. FADD acts as an adaptor to relay apoptotic signals initiated by death receptors such as Fas. FADD amplification was found to correlate with gender distinction, possibly accounting for the observed twofold increased frequency of oral cancer occurrence in males compared with females. Overexpression of FADD is more likely to make the carcinoma metastatic. *ORAOV1* (Oral Cancer Overexpressed 1) is another oncogene located at this locus, whose gain of function has been attributed to the inhibition of apoptosis, progression through the cell cycle and angiogenesis [94, 95]. Whole-exome sequencing of 120 OSCCs from male individuals in Taiwan has identified several mutational signatures [96]. Novel driver genes, such as *CHUK* and *ELAVL1*, have been identified that are involved in promoting or repressing the functions of various oncogenes and tumour suppressor genes. Two important genes mutated less frequently are *ASXL1* (coding for a transcription factor) and *RPTN* (related to epithelial differentiation). A significant group of genes involved in cell cycle regulation, including *TP53*, *CDKN2A*, and *CCND1*, were also present. Loss of the*TP53* allele has long been associated with defects in cell cycle regulation, leading to uncontrolled amplification [97]. Tumours predominantly located in the tongue could be targeted by drugs against the*TP53* and *CCND1* genes of the p53-cell cycle pathway. More than 50% of the tumours were found to carry at least one aberrant event potentially targeted by US Food and Drug Administration approved agents.

Gingivo-buccal oral squamous cell carcinoma (OSCC-GB) is a clinical subtype of OSCC, prevalent in the Asian sub-continent, where tobacco chewing is common. A synergistic contribution of related major OSCC driver genes, such as *MAP4K2*, *FAT1*, *EPHA2*, *NOTCH1*, *CASP8*, *HRAS*, *RASA1*, and *PIK3CA*, has been found to contribute to tumourigenesis, with the most common anatomical site being the buccal mucosa. Genetic signatures indicate that mutations in several cancer genes, including *USP9X*, *MLL4*, *ARID2*, *UNC13C* and *TRPM3*, are specific to OSCC-GB [96, 98]. These specific genes are functionally involved in the suppression of tumours. Some genetic markers, such as *TP53*, *FAT1*, *CASP8*, *HRAS* and *NOTCH1*, are shared between OSCC-GB and HNSCC. Alterations in some new genes, for example, *DROSHA*, *YAP1* and *DDX3X*, have been discovered in OSCC-GB. An enrichment of alterations in Wnt signalling, dorso-ventral axis formation and axon guidance pathways was also discovered. A very high proportion of C4G mutations, especially among tobacco users, was observed by the India Project Team of the International Cancer Genome Consortium [98]. It seems that mutations in tumour suppressor genes are more prevalent than mutations of oncogenes in oral cancer. Some of the important genetic markers for oral cancer are listed in Table 2.

**Table 2:** List of some genes, which have been found to play a significant role in the development of oral carcinoma.

Gene	Locus	Function	Reference
*CCND1*	11q13	Cell cycle regulation	[72, 76, 77, 78, 79, 86]
*CTTN*	11q13	F-actin binding protein	[72, 74, 75]
*FADD*	11q13	Regulation of apoptosis	[83]
*ORAOV1*	11q13	Regulation of apoptosis and angiogenesis	[84, 85]
*TP53*	17p13	Regulation of cell cycle and apoptosis	[87]
*FAK/PTK2*	8q23–24	Cellular adhesion and spreading	[74]
*PIK3CA*	3q26	Regulation of receptor- mediated extracellular stimuli	[86]
*SOX2*	3q26	Transcription factor	[86]
*EGFR*	7p12	Receptor for epidermal growth factor protein ligands	[90, 91, 92]

### 2.6 Gene expression studies and the role of HPV

Gene expression analyses can be used to predict lymph node metastasis and the extracapsular spread of metastatic nodes in patients of OSCC [99]. *CTTN* and *MMP9* have been found to be two important biomarkers whose overexpression can be used to predict the lymph node metastasis and extracapsular spread. *MMP9* is a zinc metalloprotease that can degrade the collagen of the extracellular matrix. Other candidates are *BMP2*, a member of the transforming growth factor-β superfamily, and epidermal growth factor receptor (*EGFR*). Fluorescence in situ hybridization (FISH) and immunohistochemistry (IHC) studies on OSCC samples by Martin-Ezquerra et al. have detected genetic abnormalities in a set of cell cycle markers, including*TP53*, *MYC*, *CCND1*, *ERBB2* and *EGFR* [100]. As expected, loss of the tumour suppressor gene *TP53* and copy number gains of the oncogenes *MYC*, *CCND1*, *ERBB2* and *EGFR* were detected in tumoural lesions. These same genetic abnormalities were infrequent in precursor lesions and totally absent in inflammatory lesions. Thus, evaluation of these genes can act as a diagnostic tool to distinguishing benign from malignant oral lesions. Since, multiple genetic alterations are involved in the pathogenesis of OSCC, monoclonal antibody targeted therapies are unlikely to be very effective.

A CGH study on primary OSCCs obtained from 97 patients by Pathare et al. [101] identified several genomic alterations caused mainly by tobacco use. A strong correlation of 7p gain and 8p loss with node-positive OSCC was observed. Activation of the oncogene *EGFR* on 7p12 and loss of the tumour suppressor genes on 8p may contribute to the lymph node involvement of primary OSCC. Loss of the q arm of chromosome 18 was also found to decrease survival. Their findings revealed the importance of simultaneous 11q13 gain and 18q loss as a predictor of poor prognosis. An array comparative genomic hybridization analysis of progressive oral potential malignant lesions and same-site OSCCs identified copy number alterations that may be associated with disease progression [102]. A majority of them were amplifications observed on chromosomes 1p, 11q13.4, 9q34.13, 21q22.3, 6p21, 6q25, 10q24, 19q13.2, 22q12, 5q31.2, 7p13, 10q24 and 14q22. DNA losses were observed at a lesser frequency on 5q31.2, 16p13.2, 9q33.1, 9q33.29, 17q11.2, 3p26.2, 18q21.1, 4q34.1 and 8p23.2. Some of the associated genes identified and validated by quantitative real-time polymerase chain reaction are *BTBD7*, *KHDRBS1*, *PARP1* and *RAB1A*, indicating changes in transcriptional and post-transcriptional control mechanisms as important factors for disease progression. By combining array-CGH with mRNA expression and tissue microarray analysis, Freier et al. found *SOX2* in 3q26.33 coding for a transcription factor and *CCNE1* in 19q12, a member of the cyclin family, as important candidate genes on OSCC specimens [103]. Using array-based comparative genomic hybridization on microdissected OSCCs, Chen et al. have observed copy number gains in *TP63*, *Serpine1*, *FGF4/FGF3*, *c-Myc*, *EGFR*, *CCND1*, *EMS1*, *AIB1* and *DMD* genes clustering mainly on 3q27–29, 7p, 17q21–tel, 11q13, 20q and the X chromosome [104]. Among them, gains of *EGFR* at 7p, *FGF4/FGF3*, *CCND1*and *EMS1*at 11q13, and *AIB1* at 20q were significantly associated with lymph node metastasis. On the other hand, deletions were detected for *Caspase8* and *MTAP*. An areca quid (AQ) chewing habit linked to Asian countries differentially affect the genomic profiles of *FHIT* at 3p14.2 and *EXT1* at 8q24.11-13.

It is very important to differentiate between HPV-related and HPV-unrelated OSCC because of competing treatment strategies. The number of chromosomal alterations to form tumours is lower in the presence of HPV due to the inactivation of the tumour suppressor proteins p53 and pRb by the viral E6 and E7 oncoproteins, respectively. Copy number gain at 3q26.3 and 11q13 was present irrespective of HPV status, although its effect was more severe in HPV-negative tumours [105, 106]. OSCC in the presence of HPV showed significantly more losses at chromosomes 3p, 5q, 9p, 15q, and 18q and, less often, Xp gains. Loss of 16q in HPV-related OSCC was found to be a strong indicator of favourable outcome with no recurrence. This information should be considered to improve treatment decisions.

**Fig. 1:**
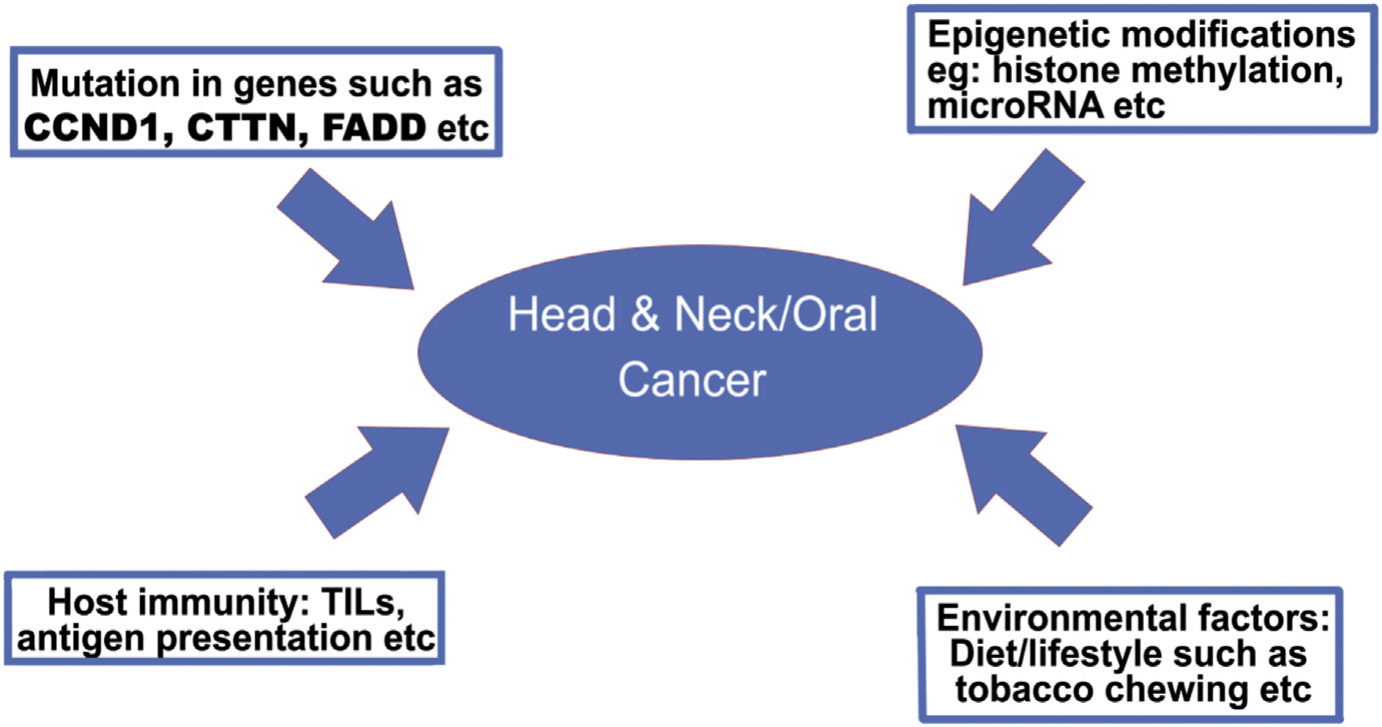
Various factors contributing to the occurrence of head and neck/oral carcinoma. TILs: Tumour infiltrating lymphocytes.

## 3 Conclusions

Among the various categories of HNSCC, cancer of the oral cavity is the most common malignancy and has a high morbidity rate (37.8%) five years after diagnosis [107, 108]. With late detection and poor prognosis, OSCC has been the most relevant epithelial malignancy for dental surgeons [109]. OSCC is a highly heterogeneous, complex subtype of cancer [110]. Tobacco, alcohol, viruses and diet are several of the risk factors involved. These factors, along with genetic inheritance, may have a carcinogenic effect on the respiratory and digestive system's normal cells. This can occur in regions such as the mouth, and the tongue, lower lip and mouth floor are the most affected regions [107, 111].

This review article sheds light on the role of various immune factors involved in HNSCC and the genetic markers responsible for the development of OSCC (Fig. 1). As seen from this review, several genetic signatures specific to OSCC are available. In contrast to immunological markers, a plethora of research work has been directed towards the broader purview of HNSCC, but the same data specific for OSCC are very limited. That is exactly where our group is planning to contribute in the near future. A histological analysis of tumours from the oral cavity has shown a correlation between increased intratumoural or stromal TILs and a better prognosis [112]. The identification of genetic abnormalities is becoming easier with the rapid progress in detection technologies. Emerging technologies that can provide both genetic and epigenetic information have the potential to provide an overall picture of tumour progression. Recently, a group from China reported a set of twenty-four genes that are postulated to be important for the classification and prognosis of OSCC [113].

Along with genomic aberrations, regulation of the immune profile and the interplay between them can also have a significant effect on the pathological features and treatment outcome of a particular patient. An example of this interplay can be seen in the loss or down-regulation of HLA genes belonging to the MHC on chromosome 6 that provides tumour cells an opportunity to evade the host immune response [114, 115]. Recognition of TAA by HLA-restricted T cells is fundamental for the detection and destruction of malignant cells [116]. Alterations in MHC can be reversible regulatory defects or irreversible structural defects that can influence the outcome of cancer immunotherapy [117]. When there is a loss of expression due to transcriptional regulation, it can be reversed by cytokine treatment, and T-cell-based therapy can be successfully applied. However, immunotherapy aimed at augmenting a T-cell-specific anti-tumour response may not be effective in cases of structural damage to HLA genes that is irreversible. Therefore, immunotherapy needs to include precise identification of the HLA genotype. Analysis of HLA-A, B, and C genes on microdissected tumours correlated loss of expression to metastasis [118]. Loss of heterozygosity on chromosome 15, which codes for β-2 microglobulin, is quite prevalent in some cancers [119]. This could represent one of the early events in malignant cells showing a normal HLA-pattern, leading to the generation of precommitted tumours to become HLA escape variants. Another example of the integrity between genomic alterations and immunomodulation in many different types of cancer can be observed in copy number gains of chromosome 9p involving PD-L1 [120]. Apart from PD-L1, chromosome 9p also harbours PD-L2 and the Janus Kinase 2 gene (JAK2) adjacent to one another and localized exactly to 9p24.1. JAK2 is a transcriptional activator of both PD-1 ligands and amplification of this locus provides a distinct molecular subtype in breast cancer, gastric cancer and lymphomas [121, 122, 123, 124]. The PDJ amplicon in tumours seems to represent a biomarker that can be used to advance personalized therapies for cancer patients. The genetic basis of PD-1 ligand deregulation and overexpression in Hodgkin's lymphoma suggests that a blockade with an anti-PD-1 antibody could be used to treat this disease. In fact, Ansell et al. have shown that Nivolumab (a PD-1 blocking antibody) can be used as a therapeutic agent in patients suffering from relapsed Hodgkin's lymphoma [125].

It is quite obvious that increased neoantigen diversity caused by genomic alterations will have an effect on the immune infiltrate within TME. Future research related to both the positive and negative effects of gene amplification and the effectiveness of combined therapies that target both genetic and immune markers is warranted. One of the main challenges will be to integrate the staggering amount of data that is being generated in cancer research for better management.

## Declarations

### Author contribution statement

All authors listed have significantly contributed to the development and the writing of this article.

### Funding statement

This work was supported by Department of Biotechnology, India.

### Competing interest statement

The authors declare no conflict of interest.

### Additional information

No additional information is available for this paper.
